# Ferulic Acid Alleviates Lipid and Bile Acid Metabolism Disorders by Targeting FASN and CYP7A1 in Iron Overload-Treated Mice

**DOI:** 10.3390/antiox13111277

**Published:** 2024-10-23

**Authors:** Yaxu Liang, Jun Qi, Dongming Yu, Zhibo Wang, Weite Li, Fei Long, Shuai Ning, Meng Yuan, Xiang Zhong

**Affiliations:** 1 College of Animal Science and Technology, Nanjing Agricultural University, Nanjing 210095, China; 2021205036@stu.njau.edu.cn (Y.L.); 2023105089@stu.njau.edu.cn (J.Q.); 2022205042@stu.njau.edu.cn (D.Y.); 2020205010@stu.njau.edu.cn (Z.W.); 2023205049@stu.njau.edu.cn (W.L.); 2022105065@stu.njau.edu.cn (F.L.); 2023805168@stu.njau.edu.cn (S.N.); 2023105090@stu.njau.edu.cn (M.Y.); 2 Natural Plant and Animal Health Innovation Institute, NJAU-Cohoo Biotechnology, Nanjing Agricultural University, Nanjing 210095, China

**Keywords:** iron overload, ferulic acid, lipid metabolism, bile acid metabolism, FASN, CYP7A1

## Abstract

Iron overload is a common complication in various chronic liver diseases, including non-alcoholic fatty liver disease (NAFLD). Lipid and bile acid metabolism disorders are regarded as crucial hallmarks of NAFLD. However, effects of iron accumulation on lipid and bile acid metabolism are not well understood. Ferulic acid (FA) can chelate iron and regulate lipid and bile acid metabolism, but its potential to alleviate lipid and bile acid metabolism disorders caused by iron overload remains unclear. Here, in vitro experiments, iron overload induced oxidative stress, apoptosis, genomic instability, and lipid deposition in AML12 cells. FA reduced lipid and bile acid synthesis while increasing fatty acid β-oxidation and bile acid export, as indicated by increased mRNA expression of *PPARα*, *Acox1*, *Adipoq*, *Bsep*, and *Shp*, and decreased mRNA expression of *Fasn*, *Acc*, and *Cyp7a1*. In vivo experiments, FA mitigated liver injury in mice caused by iron overload, as indicated by reduced AST and ALT activities, and decreased iron levels in both serum and liver. RNA-seq results showed that differentially expressed genes were enriched in biological processes related to lipid metabolism, lipid biosynthesis, lipid storage, and transport. Furthermore, FA decreased cholesterol and bile acid contents, downregulated lipogenesis protein FASN, and bile acid synthesis protein CYP7A1. In conclusion, FA can protect the liver from lipid and bile acid metabolism disorders caused by iron overload by targeting FASN and CYP7A1. Consequently, FA, as a dietary supplement, can potentially prevent and treat chronic liver diseases related to iron overload by regulating lipid and bile acid metabolism.

## 1. Introduction

Iron is an indispensable trace element in the body, which plays important roles in numerous biological processes such as DNA and RNA biosynthesis and repair, energy generation, signal transduction, and immune defense [1]. However, excessive iron deposition can lead to severe cytotoxicity and tissue damage. Notably, iron overload is a common complication in patients with non-alcoholic fatty liver disease (NAFLD) [2,3]. Lipid metabolism disorder is regarded as a crucial hallmark of NAFLD [4,5]. Therefore, to prevent and treat NAFLD, it is crucial to study the effect of iron accumulation on lipid metabolism and perform potential nutritional interventions to mitigate the lipid metabolism disorders and hepatic damage resulting from iron overload.

Phenolic compounds exhibit protective roles against cancer, neurodegenerative diseases, heart diseases, and inflammation through their metal chelation and free radical scavenging abilities [6]. Ferulic acid (FA), the most abundant phenolic acid in cereal grains, is known for its high bioavailability [7]. Noteworthy, FA possesses iron-chelating capacity [8] and exhibits various physiological functions, including anticancer, anti-inflammatory, antioxidant, antimicrobial, and hepatoprotective [9,10]. Crucially, FA can regulate lipid metabolism. Administration of ferulic acid (FA) at doses of 30 or 60 mg/kg body weight per day in rats alleviated metabolic syndrome induced by a high-fat diet, resulting in lower serum triglyceride (TG) and total cholesterol (TC) levels [11]. FA could decrease lipid accumulation by regulating the AMPKα/SREBP1/ACC1 pathway or inhibiting the expression of the extracellular signal-regulated kinase (ERK)1/2, c-Jun amino-terminal kinase (JNK)1/2/3, and HGMB1 [12,13]. Moreover, FA can improve fat-induced hyperlipemia by modulating the lipogenic enzyme activities and increasing the lipid excretion [14]. However, whether ferulic acid can alleviate lipid metabolism disorders induced by iron overload remains unclear.

Notably, lipid and bile acid metabolism are closely intertwined [15,16]. Bile acids are the metabolic products of cholesterol. Lipids can regulate bile acid metabolism [15]. Fatty acid synthase (FASN) plays a crucial role in lipid metabolism by catalyzing the de novo synthesis of fatty acids from acetyl-CoA and malonyl-CoA. Excessive dietary lipid promoted TC deposition by upregulating FASN, further elevating the content of secondary bile acids in feces [16]. In contrast, bile acids can regulate lipid metabolism through cholesterol 7-alpha-hydroxylase (CYP7A1), which is the rate-limiting enzyme for bile acid synthesis, thereby affecting cholesterol content [17,18]. CYP7A1 deficiency in humans can lead to hypercholesterolemia, further underscoring the pivotal role of this enzyme in lipid homeostasis [19]. Previous studies have shown FA could attenuate high-fat diet-induced hypercholesterolemia by activating CYP7A1 to promote bile acid synthesis [20]. Given the interplay between lipid and bile acid metabolism, as well as FA’s iron-chelating and lipid-regulating properties, it is of paramount importance to investigate whether FA can alleviate lipid and bile acid metabolism disorders caused by iron overload.

## 2. Materials and Methods

### 2.1. Cell Culture

AML-12 (alpha mouse liver 12) cells were purchased from YIFEIXUE Biological Technology, Nanjing, China (Cat No. CL-0602). Cells were cultured in DMEM/F12 medium (Cat No. 11320033, Gibco, Carlsbad, CA, USA), supplemented with 10% fetal bovine serum (FBS), 1% penicillin/streptomycin, 1% insulin-transferrin-selenium (ITS), and 0.1% dexamethasone. Upon reaching 70% confluence, the cells in ferric ammonium citrate (FAC) group were treated with 25 µM 8-hydroxyquinoline (8-Hyd) and 40 µM FAC. The cells in ferulic acid (FA) group were treated with 25 µM 8-Hyd, 40 µM FAC, and 5 µM FA for 24 h.

### 2.2. Mice

Mouse experiments were conducted following guidelines authorized by the Animal Ethics Committee of Nanjing Agricultural University (Permit number SYXK-2017-0007). The mice used were male with a C57BL/6J genetic background.

Thirty mice, aged 6–7 weeks and weighing 20–25 g, were randomly divided into three groups: normal group (NC), iron overload group (Fe), and iron overload + ferulic acid group (FA). The mice in the NC group were fed a basal diet containing 0.045% iron for 2 months, while the diets of the Fe and FA groups were supplemented with 3% carbonyl iron added to the basal diet. At the second month, mice in the FA group were orally administered with 50 mg/kg body weight/day of ferulic acid dissolved in sodium carboxymethyl cellulose solution (CMC-Na) through intragastric gavage, while the remaining two groups were given an equal amount of CMC-Na solution four times a week. Mice were placed in a room with a temperature of 22 ± 0.5 °C, relative humidity of 50 ± 5%, and a 12 h light/dark cycle. Mice had ad libitum access to rodent diet and water. Mice were fasted for 12 h prior to slaughter.

### 2.3. Cell Viability Assay

The cell counting kit-8 (CCK-8) experiment was performed to assess cell viability following manufacturer’s protocol (BS350B, Biosharp, Nanjing, China).

### 2.4. Detection of Activities of Aspartate and Alanine Aminotransferase, and Iron Content in Serum

Activities of aspartate and alanine aminotransferase (AST/ALT, Cat No. C010-2-1, C009-2-1) and iron content (Cat No. A039-1-1) in serum were determined using kits from Jiancheng Company, Nanjing, China, following the manufacturer’s protocols.

### 2.5. Detection of Triglyceride, Total Cholesterol, Total Bile Acid, Malondialdehyde, and Iron in Tissues or Cells

Triglyceride (TG), total cholesterol (TC), total bile acid (TBA), malondialdehyde (MDA), and iron contents were quantified using kits in strict accordance with the manufacturer’s instructions (Maibo Biotechnology Co., LTD, Nanjing, China). The specific kit item numbers utilized were A110-1-1 for TG, A111-1-1 for TC, E003-2-1 for TBA, A003-2 for MDA, and A039-1-1 for iron.

### 2.6. Detection of Reactive Oxygen Species (ROS) in Cells

Cells were collected after being treated with 8-Hyd and FAC for 24 h. To assess the level of ROS, the cells were suspended in 10 μmol/L 2′,7′-dichlorofluorescin diacetate (DCFH-DA) and incubated at 37 °C for 20 min. After incubation, the cells were washed three times with serum-free cell medium. Subsequently, the cells were centrifuged at 1500 rpm for 5 min to pellet the cells. The supernatant was discarded, and the cells were resuspended in phosphate-buffered saline (PBS). The cell suspension was then transferred to a 96-well plate and detected using a fluorescence microplate reader with an excitation wavelength of 488 nm and the emission wavelength of 525 nm.

### 2.7. mRNA Expression Analysis

Total RNA was extracted from liver tissues or cells with Trizol reagent (Invitrogen, Carlsbad, CA, USA). The liver samples and cells were resuspended with 1 mL of pre-cooled Trizol reagent. A total of 200 μL of trichloromethane solution was added, and the mixture was incubated for 5 min to promote the separation of organic and aqueous phases. After centrifugation at 4 °C and 12,000× *g*/min for 15 min, the supernatant was transferred into a new enzyme-free centrifuge tube and incubated with an equal volume of pre-cooled isopropyl alcohol for 10 min. After centrifugation at 4 °C and 12,000× *g*/min for 10 min, the supernatant was discarded, and the precipitate was washed twice with pre-cooled 75% ethanol solution. Finally, RNA was dissolved in RNase-free water. The total RNA concentration and purity were determined by Thermo NanoDrop 2000 (Thermo Fisher, Waltham, MA, USA).

The RNA was reverse-transcribed into cDNA according to the instructions of the reverse transcription kit (Cat No. RR047A, TaKaRa Biotechnology Co., Ltd., Dalian, China). The qRT-PCR experiment was performed using SYBR (Accurate Biotechnology, Cat No. 11718, Changsha, China). The total reaction volume was 10 μL. The reaction was carried out according to the following procedure: predenaturation stage at 95 °C for 5 min; cyclic reaction stage at 95 °C for 10 s, 60 °C for 30 s, and the number of cycles was 40; melting curve stage at 95 °C for 15 s, 60 °C for 1 min, and 95 °C for 15 s. Primers were designed using NCBI, and *GAPDH* was the internal reference gene. Primer sequences and accession numbers of genes were presented in Table S1.

### 2.8. Western Blot

The total protein was extracted using a radio-immunoprecipitation assay (RIPA). The protein concentration was quantified using BCA kit (Cat No. P0010, Biyuntian Biotechnology, Nanjing, China) according to the instructions. Protein was diluted to 1 μg/µL and denatured with 5×SDS-PAGE loading buffer at 100 °C for 5 min. The Western blot procedure was as follows: the total protein was separated by sodium dodecyl sulphate-polyacrylamide gel electrophoresis (150 V, 45 min). Protein was transferred to a polyvinylidene fluoride membrane at 300 mA for 45 min. The 5% skim milk powder solution was used to block membrane for 2 h at room temperature. The antigen was incubated with the primary antibody at 4 °C for 12 h, followed by incubation with secondary antibody at room temperature for 1 h. The protein was visualized under an ultra-sensitive chemiluminescent gel imaging instrument and was quantitatively analyzed by ImageJ software, https://imagej.net/ij/, (NIH, Baltimore, MD, USA). Details of antibodies were presented in Table S2.

### 2.9. Hematoxylin Eosin Staining

After the liver samples were fixed in 4% paraformaldehyde for 24 h, they were immersed in 70%, 85%, 95%, anhydrous ethanol, and xylene to undergo dehydration and transparentization. Subsequently, the tissue was embedded in paraffin wax and sectioned into 5 μm thick slices. For hematoxylin and eosin staining, liver sections were deparaffinized and rehydrated in xylene, anhydrous ethanol, 90%, 80%, 70%, and 50% ethanol solution. The nucleus was stained with hematoxylin, and the cytoplasm was stained with eosin. The morphology of liver tissue sections was observed using an inverted optical microscope (Nikon ECLIPSE 80i, Nikon Corporation, Tokyo, Japan).

### 2.10. Immunofluorescence

The cells were fixed in 4% paraformaldehyde solution at room temperature for 15 min and then infiltrated in 0.3% Triton X-100 (ST797, Beyotime, Nanjing, China) for 20 min. To reduce nonspecific binding, cells were incubated with 3% bovine serum albumin for 60 min. Next, the cells were incubated with the primary antibody at 4 °C for 12 h, followed by incubation with secondary antibody from the Alexa series at room temperature for 1 h. Then, the nucleus was stained using DAPI (Cat No. BL105A, Biosharp, Nanjing, China). The images were captured and quantitatively analyzed using an LSM710 confocal laser scanning microscope (LSM 700-Zeiss, Zeiss Corporation, Jena, Germany) and ImageJ software.

### 2.11. Flow Cytometry Analysis of Cell Cycle

Cells were fixed in 70% alcohol at −20 °C for 12 h. Subsequently, they were stained with 0.025 mg/mL propidium iodide for 10 min using the Cell Cycle Detection Kit (KeyGen Biotech, Nanjing, China) following the manufacturer’s instructions. Data analysis was performed using FlowJo 10 software.

### 2.12. RNA-Seq

Total RNA was isolated from liver tissue using Trizol reagent (Invitrogen, Carlsbad, CA, USA), followed by assessment of RNA quality and integrity using NanoDrop 2000 spectrophotometry and Agilent 2100 (Agilent Technologies Inc., Santa Clara, CA, USA), respectively. Messenger RNA (mRNA) containing PolyA was isolated using Dynabeads Oligo (dT)_25_ (Invitrogen, Carlsbad, CA, USA). Subsequently, mRNA was fragmented into 150–200 bp using an ultrasonic processor (Diagenode, Danville, NJ, USA), with each cycle consisting of 30 s on and 30 s off, for a total of 30 cycles. Library preparation was conducted using the TruSeq RNA Sample Preparation Kit (Illumina, San Diego, CA, USA), following the manufacturer’s protocol. The prepared libraries were then analyzed using the Illumina Hiseq X-ten platform. Detailed protocols and procedures for data analysis were provided by GENE DENOVO (Guangzhou, China).

### 2.13. Statistical Analysis

The experiments were repeated at least three times, and the data were presented as the mean ± standard error of the mean (SEM). SPSS 26.0 software (IBM, Armonk, NY, USA). was used for statistical analysis, and GraphPad Prism 8.4.2 software (San Diego, CA, USA) was used for graphing. Two-tailed Student’s *t*-test was used to analyze the differences between NC and FAC groups, FAC and FA groups, NC and Fe groups, and Fe and FA groups. *p* < 0.05 was considered statistically significant. In the CCK8 assay, data were analyzed by one-way analysis. The difference was significant when data had different letters (*p* < 0.05), whereas there was no significant difference when data had the same letters (*p* > 0.05).

## 3. Results

### 3.1. Construction of Iron Overload Model in AML12 Hepatocytes

**Figure 1:**
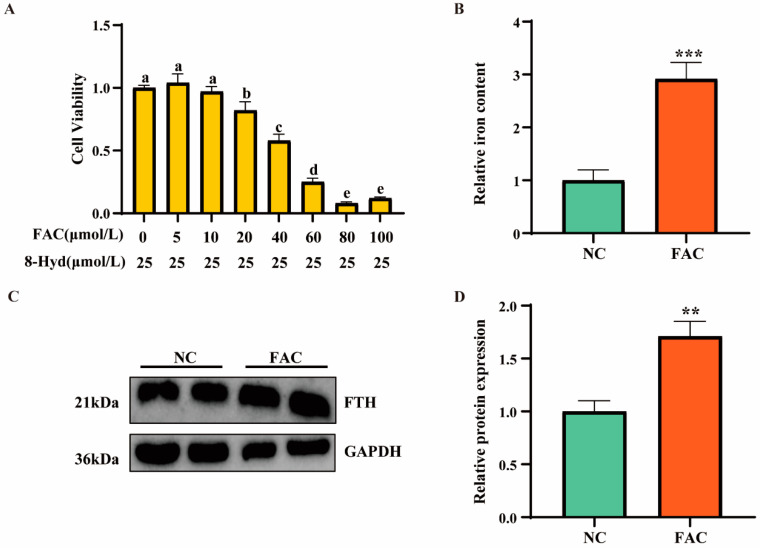
Iron overload model in AML12 hepatocytes was created. (**A**) Cell viability was detected by CCK8 assays in AML12 cells treated with ferric ammonium citrate (FAC) and 8-hydroxyquinoline (8-Hyd). (**B**) Relative iron content in AML12 cells treated with or without FAC. (**C**,**D**) Protein expression level of ferritin heavy chain (FTH) was detected by Western blot. In (**A**), statistical significance among groups was denoted by different letters (*p* < 0.05). Groups sharing the same letter were not significantly different from each other (*p* > 0.05). Statistical significance was also indicated as ** *p* < 0.01, *** *p* < 0.001 (**B**–**D**). “NC” represents “negative control group”. In NC group, AML12 cells were treated with 25 μM 8-hydroxyquinoline (8-Hyd). This served as a baseline for comparison with the FAC group, where AML12 cells were treated with 25 μM 8-Hyd and 40 μM FAC.

To create an iron overload condition in hepatocytes, mouse hepatocytes AML12 were incubated with 8-hydroxyquinoline (8-Hyd) and different concentrations of ferric ammonium citrate (FAC). FAC is an extremely stable form of ferric citrate in blood. 8-Hyd is a lipophilic chelator that promotes rapid entry of iron into cells [21]. The combination of FAC and 8-Hyd has been widely used in vitro to induce iron overload [21]. As shown in Figure 1A, incubating AML-12 cells with 25 µM 8-Hyd and 40 µM FAC significantly reduced cell viability to 58%, but 60 µM FAC reduced cell viability to less than 50%. Moreover, the combination of 25 µM 8-Hyd and 40 µM FAC significantly increased iron content and protein expression level of iron storage protein ferritin heavy chain (FTH) (Figure 1B–D). Therefore, this study chose 25 µM 8-Hyd and 40 µM FAC to construct an iron overload model for the follow-up experiments.

### 3.2. Iron Overload Induced Oxidative Stress, Apoptosis, and Genomic Instability in AML12

To evaluate effects of iron overload on cell injury, we analyzed oxidative stress, apoptosis, and genomic instability. Additionally, this study detected cell cycle by flow cytometry. Compared with the control group, iron overload significantly upregulated ROS content and mRNA expression levels of *Bax* and *Caspase3* (Figure S1A,B). Iron overload significantly downregulated protein levels of KEAP1, HO-1, and BCL2, while upregulating the BAX/BCL2 ratio (Figure S1C–E). Oxidative stress and apoptosis can promote genomic instability to exacerbate damage [22]. γH2A.X is a sensitive molecular marker of DNA damage [23]. PARP1 could quickly sense DNA damage and recruit repair machinery to damage sites to maintain genomic stability [24]. PRDX2 can regulate replication fork speed to protect cells against oxidative stress and DNA damage [25]. As shown in Figure S1F,G, compared with the control group, FAC significantly downregulated PARP1 and PRDX2 protein expression. Immunofluorescence revealed FAC significantly increased the percentage of γH2A.X positive cells (Figure S1H). Flow cytometry showed iron overload decreased the cell number in G1 phase and increased the cell number in G2 phase (Figure S2). The above results suggested that iron overload led to cell oxidative stress, apoptosis, and genomic instability, resulting in cell damage.

### 3.3. Iron Overload Caused Lipid and Bile Acid Metabolism Disorders in Hepatocytes

**Figure 2:**
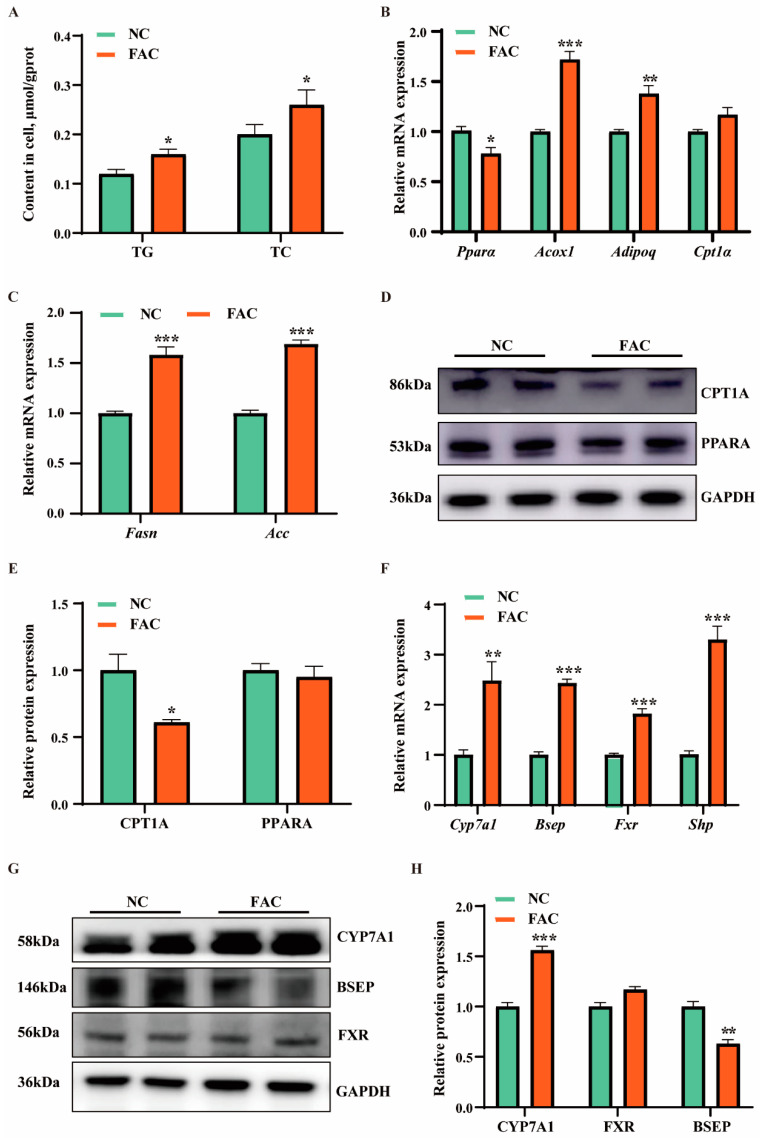
Effects of iron overload on lipid and bile acid metabolism in AML12. (**A**) The contents of triglycerides (TG) and total cholesterol (TC) in AML12 cells treated with or without FAC. (**B**,**C**) qRT-PCR analysis of expression of lipolysis (**B**) and lipogenesis (**C**)-related genes. (**D**,**E**) Western blot analysis of expression of lipid metabolism-related proteins. (**F**) qRT-PCR analysis of expression of bile acid metabolism-related genes. (**G**,**H**) Western blot analysis of expression of bile acid metabolism-related proteins. Statistical significance was indicated as * *p* < 0.05, ** *p* < 0.01, *** *p* < 0.001, determined using a two-tailed *t*-test.

To analyze effects of iron overload on lipid and bile acid metabolism in AML-12 cells, we detected contents of TG and TC, as well as expression levels of lipolysis, lipogenesis, and bile acid metabolism-related genes and proteins. As shown in Figure 2A–C, compared with the control group, iron overload significantly increased TG and TC contents, upregulated the expression levels of lipolysis-related genes (*Acox1* and *Adipoq*), and lipogenesis-related genes (*Fasn* and *Acc*). Moreover, iron overload significantly decreased the protein expression level of CPT1A (Figure 2D,E). Meanwhile, iron overload significantly upregulated bile acid metabolism-related genes (*Cyp7a1*, *Bsep*, *Fxr*, and *Shp*) and increased bile acid synthesis protein CYP7A1 expression level, but decreased bile salt export protein BSEP expression level (Figure 2F–H). Collectively, these results indicated that iron overload leads to lipid and bile acid deposition in AML12 cells.

### 3.4. Effects of Ferulic Acid on Lipid and Bile Acid Metabolism Disorders Induced by Iron Overload in AML12

**Figure 3:**
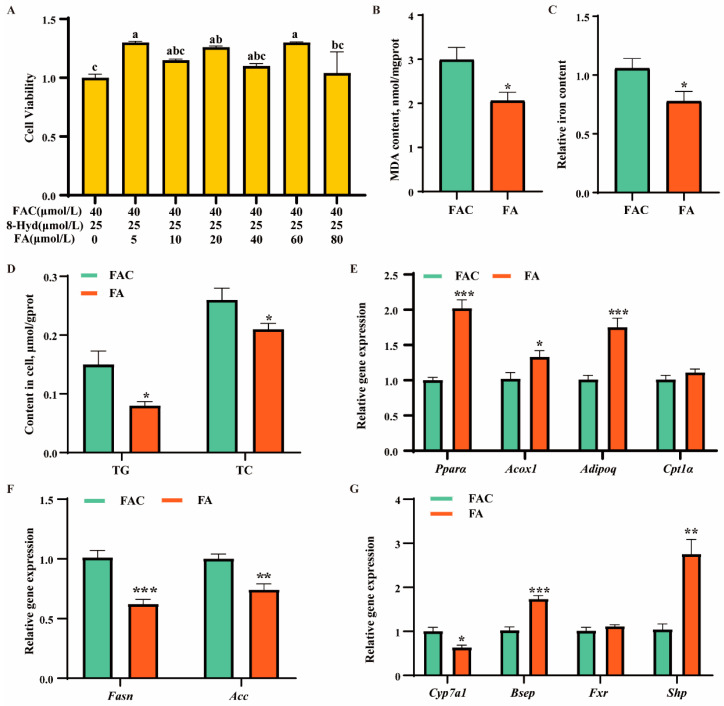
Effects of ferulic acid on lipid and bile acid metabolism in AML12 treated by FAC. (**A**) Cell viability was detected by CCK8 assays in AML12 cells treated with ferric ammonium citrate (FAC), 8-hydroxyquinoline (8-Hyd), and different concentrations of ferulic acid. (**B**) MDA content in AML12 cells induced by FAC with or without ferulic acid. (**C**) Relative iron content in AML12 cells induced by FAC with or without ferulic acid. (**D**) The contents of triglycerides (TG) and total cholesterol (TC) in AML12 cells induced by FAC with or without ferulic acid. (**E**,**F**) qRT-PCR analysis of expression of lipolysis (**E**) and lipogenesis (**F**)-related genes. (**G**) qRT-PCR analysis of expression of bile acid metabolism-related genes. In (**A**), statistical significance among groups was denoted by different letters (*p* < 0.05). Groups sharing the same letter were not significantly different from each other (*p* > 0.05). Statistical significance was also indicated as * *p* < 0.05, ** *p* < 0.01, *** *p* < 0.001 (**B**–**G**).

This study selected the optimal dose of ferulic acid (FA) using the CCK8 assay. As shown in Figure 3A, 5 μM, 20 μM, and 60 μM FA significantly enhanced cell viability compared with the iron overload group. To minimize potential cytotoxicity, and maintain both cost effectiveness and research efficiency, we chose 5 μM FA for subsequent experiments.

As shown in Figure 3B–D, FA significantly reduced the levels of the oxidative product malondialdehyde (MDA), iron, TG, and TC compared with the FAC group. Additionally, FA upregulated the expression levels of lipolysis-related genes (*PPARα*, *Acox1*, and *Adipoq*) and bile acid metabolism-related genes (*Cyp7a1*, *Bsep*, and *Shp*), while downregulating lipogenesis-related genes (*Fasn* and *Acc*) expression levels (Figure 3E–G). Collectively, these results indicated that ferulic acid may relieve lipid and bile acid deposition in AML12 cells induced by iron overload.

### 3.5. Effects of Iron Overload and Ferulic Acid on Growth Performance, Serum Biochemistry, and Iron Metabolism in Mice

To explore whether FA can alleviate liver damage caused by iron overload, in vivo experiments were performed. Thirty mice, aged 6–7 weeks and weighing 20–25 g, were randomly divided into three groups: normal group (NC), iron overload group (Fe), and iron overload + ferulic acid group (FA). The mice in the NC group were fed a basal diet containing 0.045% iron for 2 months, while the diets of the Fe and FA groups were supplemented with 3% carbonyl iron added to the basal diet. At the second month, mice in the FA group were orally administered with 50 mg/kg body weight/day of ferulic acid dissolved in sodium carboxymethyl cellulose solution (CMC-Na) through intragastric gavage, while the remaining two groups were given an equal amount of CMC-Na solution four times a week.

**Figure 4:**
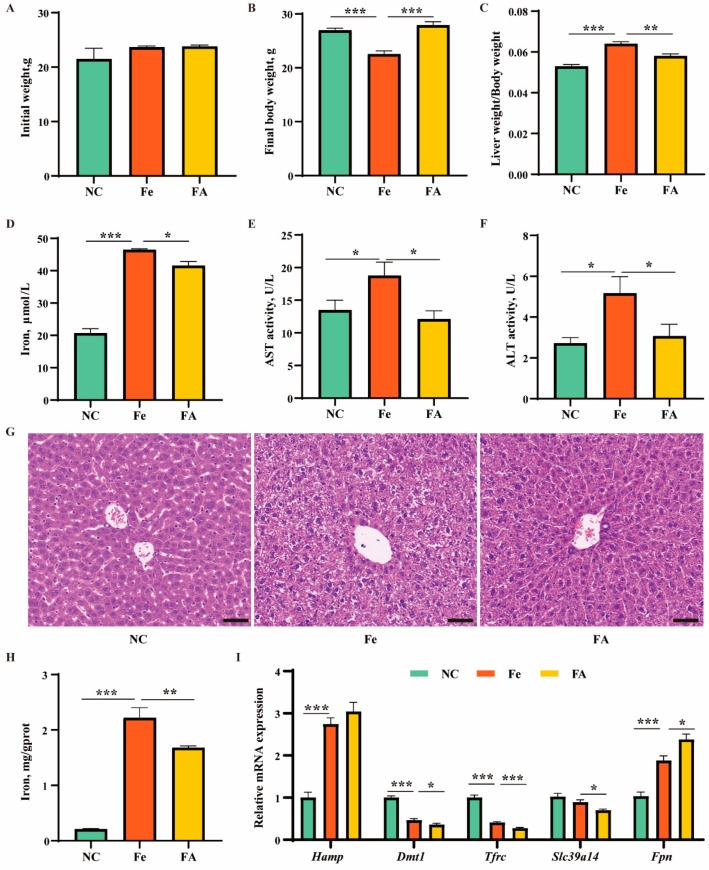
Effects of iron overload and ferulic acid on growth performance, serum biochemistry, tissue morphology, and iron metabolism. (**A**,**B**) Initial and final body weight of mice in control, iron overload, or ferulic acid supplement group. (**C**) Relative liver weight of mice in control, iron overload, or ferulic acid supplement group. (**D**–**F**) Iron content, activities of aspartate and alanine aminotransferase (AST/ALT) in serum of mice in control, iron overload, or ferulic acid supplement group. (**G**) Hematoxylin and eosin staining of mouse livers in control, iron overload, or ferulic acid supplement group. Scale bar, 50 μm. (**H**) Iron content in liver of mice in control, iron overload, or ferulic acid supplement group. (**I**) qRT-PCR analysis of expression of iron metabolism-related genes. Statistical significance was indicated as * *p* < 0.05, ** *p* < 0.01, *** *p* < 0.001, determined using a two-tailed *t*-test.

As shown in Figure 4, compared with the NC group, body weight was significantly decreased, whereas relative liver weight, iron content in serum, and activities of aspartate and alanine aminotransferase (AST/ALT) were significantly increased in the Fe group. Compared with the Fe group, FA significantly decreased relative liver weight, iron content, and activities of AST/ALT (Figure 4A–F). Hematoxylin and eosin staining showed fuzzy liver lobule structure, disordered hepatocyte cords, contracted nucleus, loose cytoplasm, and vacuolated hepatocytes in the Fe group (Figure 4G). The number of vacuolated hepatocytes was decreased in the FA group (Figure 4G). Furthermore, we examined iron content and the expression patterns of iron metabolism-related genes in livers. Compared with the NC group, iron content was significantly increased in the Fe group, while FA decreased iron level (Figure 4H). The expression levels of iron metabolism-related genes, such as *Hamp* and *Fpn*, were significantly increased in the Fe group compared with the NC group, while *Dmt1* and *Tfrc* were significantly decreased. FA further decreased mRNA expression levels of iron transporters such as *Dmt1*, *Tfrc*, and *Slc39a14* (Figure 4I). The above results indicated FA can relieve liver injury and iron metabolism disorder caused by iron overload.

### 3.6. Effects of Iron Overload and Ferulic Acid on mRNA Transcription in Mouse Livers Were Analyzed by RNA-Seq

**Figure 5:**
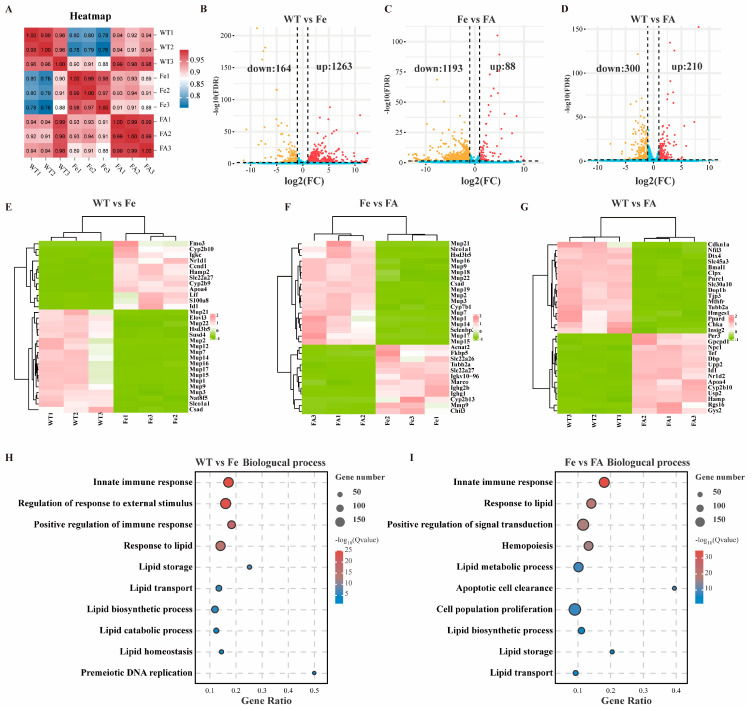
RNA-seq analyses differentially expressed genes in control, iron overload, or ferulic acid supplement group. (**A**) Pearson correlation of mouse liver in control, iron overload, or ferulic acid supplement group. (**B**–**D**) Volcano plot of differentially expressed mRNA. Red represents up-regulated genes and yellow represents down-regulated genes. (**E**–**G**) Hierarchical clustering of differentially expressed mRNA. (**H**,**I**) Top GO biological process terms enriched in differentially expressed mRNA.

**Figure 6:**
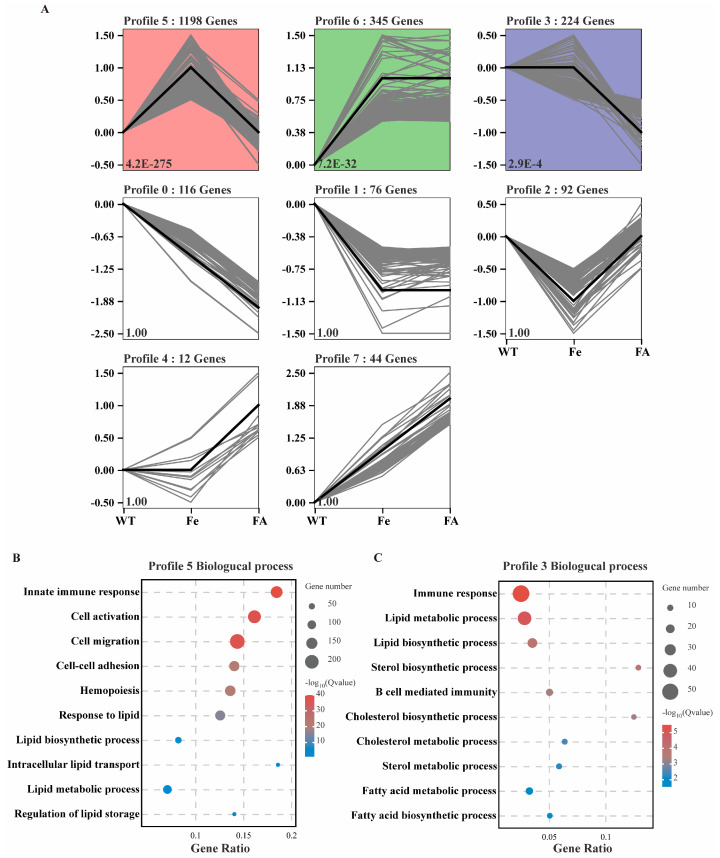
Hepatic gene expression patterns of mice in control, iron overload, or ferulic acid supplement group. (**A**) The profiles of differentially expressed genes in the mouse liver in control, iron overload, or ferulic acid supplement group. The black line represents the trend line, and the gray line represents the genes. Significantly different expression patterns are shown in a red, green, or purple background. (**B**,**C**) Top GO biological process terms enriched in differentially expressed mRNA in Profile 5 or Profile 3.

RNA-seq was performed on liver samples from 9 mice (n = 3 per group). Pearson correlation analysis showed that the sequencing results were highly repeatable (Figure 5A). Differential expression analysis identified 1427 (1263 upregulated and 164 downregulated), 1281 (88 upregulated and 1193 downregulated), and 510 (210 upregulated and 300 downregulated) differentially expressed mRNAs in the WT vs Fe (Figure 5B), Fe vs FA (Figure 5C), and WT vs FA groups (Figure 5D), respectively. Hierarchical clustering of the top 30 differentially expressed mRNAs revealed distinct patterns in their expression among different groups (Figure 5E–G). Gene ontology analysis indicated that the differentially expressed genes in the WT vs Fe (Figure 5H) and Fe vs FA (Figure 5I) groups were both associated with processes such as innate immune response, response to lipid, lipid biosynthetic process, lipid storage, and transport. To further analyze the general change trend of differential gene expression in different treatments, all differentially expressed genes were divided into 8 profiles according to their expression changes (Figure 6A). Among them, profile 5 (initially up, then down), profile 6 (initially up, then flat), and profile 3 (initially flat, then down) were significant enrichment trends (Figure 6A). Genes in profile 5 and profile 3 were enriched in biological process such as immune response and lipid metabolism (Figure 6B,C). The above studies suggested that both iron and FA had significant effects on lipid metabolism, and FA may alleviate the lipid metabolism disorder caused by iron overload.

### 3.7. Ferulic Acid Relieved Lipid and Bile Acid Metabolism Disorders Induced by Iron Overload

**Figure 7:**
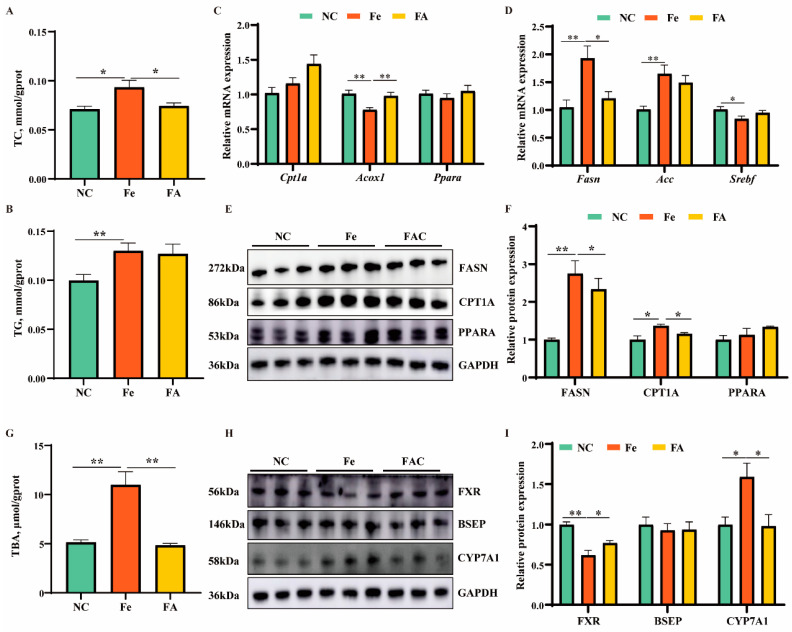
Ferulic acid relieved lipid and bile acid metabolism disorder induced by iron overload. (**A**,**B**) The contents of TG and TC in mouse liver in control, iron overload, or ferulic acid supplement group. (**C**,**D**) qRT-PCR analysis of expression of lipolysis (**C**) and lipogenesis (**D**)-related genes. (**E**,**F**) Western blot analysis of expression of lipid metabolism-related proteins. (**G**) The contents of total bile acids (TBA) in mouse liver in control, iron overload, or ferulic acid supplement group. (**H**,**I**) Western blot analysis of expression of bile acid metabolism-related proteins. Statistical significance was indicated as * *p* < 0.05, ** *p* < 0.01, determined using a two-tailed *t*-test.

Firstly, to investigate whether FA can alleviate lipid metabolism disorder induced by iron overload, we examined lipid levels in mouse livers. Iron significantly increased TC and TG contents, while FA decreased TC level (Figure 7A,B). Lipolysis-related gene *Acox1* was downregulated, while lipogenesis-related genes *Fasn* and *Acc* and FASN protein levels were upregulated in the iron overload group. FA remarkably increased expression of the *Acox1* gene while decreasing both gene and protein levels of FASN, as well as expression of the lipolysis protein CPT1A (Figure 7C–F).

Bile acids are the metabolic products of cholesterol. Hepatic bile acid synthesis is a major part of cholesterol turnover in humans [26]. Therefore, we also detected total bile acid content and expression levels of bile acid metabolism-related proteins. Compared with the control group, iron overload significantly increased total bile acid content and CYP7A1 protein level, while decreasing FXR protein expression level (Figure 7G–I). FA remarkably decreased total bile acid content and CYP7A1 protein level, while increasing FXR protein expression level (Figure 7G–I). Collectively, these results indicated FA could relieve lipid and bile acid metabolism disorders induced by iron overload.

## 4. Discussion

The liver is the major organ responsible for storing and metabolizing iron, making it extremely vulnerable to the toxicity of iron overload. Iron overload in the liver can happen in various conditions, such as hereditary hemochromatosis (a congenital disorder characterized by excessive iron accumulation), as well as conditions involving transfusions and hemolytic disorders, which are associated with iron accumulation in macrophages. Additionally, liver diseases such as hepatitis C, NAFLD, and cirrhosis are also associated with iron deposition in the liver [27]. Furthermore, lipid metabolism disorder is regarded as a hallmark of diseases [4,5]. However, the effect of iron on lipid metabolism and potential nutritional intervention for iron overload-induced liver diseases remain poorly understood. FAC is an extremely stable form of ferric citrate in the blood, while 8-Hyd is a lipophilic chelator that promotes rapid entry of iron into cells [21]. The combination of FAC and 8-Hyd has been widely used in vitro to induce iron overload [21]. AML12, a murine hepatocyte cell line, is derived from the liver of transgenic mice overexpressing transforming growth factor α [28]. AML12 cell has been widely used in studies focusing on lipid metabolism and non-alcoholic fatty liver disease [29,30,31]. In this study, treatment of AML-12 cells with 25 µM 8-Hyd and 40 µM FAC significantly increased cellular iron content and expression of the iron storage protein FTH, successfully creating an in vitro high-iron model. Dietary supplementation with carbonyl iron is commonly used to induce iron overload in mice [15]. In the present study, dietary supplementation with 3% carbonyl iron for 2 months notably elevated iron levels in both serum and liver, indicating the successful induction of an in vivo iron overload model.

Hepatic iron deposition is a significant pathological feature in approximately one-third of NAFLD patients [32]. A key feature of NAFLD is lipid accumulation due to an imbalance between lipolysis and lipid synthesis [33]. Notably, this study demonstrated iron overload significantly promoted lipogenesis in both vivo and vitro models, with upregulated expression of the lipogenesis-related gene *Fasn*. Fatty acid synthase (FASN) plays a crucial role in lipid metabolism by catalyzing the de novo synthesis of fatty acids from acetyl-CoA and malonyl-CoA. These findings aligned with previous research showing that iron supplementation promoted lipid deposition in the liver by upregulating lipogenesis-related proteins FAS and SREBP1, while downregulating fatty acid oxidation-related proteins PPARα and AOX [32]. Interestingly, we found iron overload upregulated expression of lipolysis-related genes *Acox1* and *AdipoQ* in AML12 and fatty acid oxidation protein CPT1A expression in mouse liver. This suggested a compensatory mechanism where cells attempted to upregulate lipolysis-related pathways to accelerate lipid oxidation and release, potentially counteracting elevated lipid levels caused by enhanced lipogenesis.

In addition to lipid metabolism, bile acid metabolism is also critically affected by iron overload. Bile acids are the metabolic products of cholesterol [15]. Total bile acids were shown to be significantly elevated in both livers and serum of iron overload mice induced by oral administration of ferrous sulfate, dietary supplementation of carbonyl iron, or intraperitoneal injection of iron dextran [15,34]. This dysregulation may result from downregulated bile salt export pump (BSEP) activity, which inhibits bile acid secretion [15,34]. However, in our study, iron overload did not affect BSEP expression in the mouse liver. Instead, FAC and carbonyl iron promoted bile acid deposition by upregulating the expression of bile acid synthesis-related protein CYP7A1. Accumulated bile acids are hepatotoxic, potentially leading to fatty liver, diabetes, dyslipidemia, and cardiovascular diseases [35]. Therefore, the nutrition regulation of bile acid homeostasis is crucial for liver physiology and pathophysiology.

Ferulic acid is the most abundant phenolic acid widely found in grains, fruits, and vegetables [7]. FA performs various physiological functions, including anticancer, anti-inflammatory, antioxidant, antimicrobial, and hepatoprotective [9,10]. Noteworthy, FA processes iron-chelating capacity [8]. Consistently, we found FA significantly decreased iron levels in both serum and liver of iron overload mice. This suggested that FA may alleviate iron-induced liver injury through its iron-chelating properties. FA has been shown to reduce liver steatosis in animal models of high-fat and alcohol-induced fatty liver disease [36,37]. Methyl FA could inhibit lipid synthesis through AMPK and FoxO1 signal pathways while promoting lipid oxidation via the SIRT1/PPAR-α/CPT-1α axis, ultimately attenuating ethanol-induced hepatic steatosis [36]. Fatty acid synthase (FASN), a central regulator of lipid metabolism, plays an important role in the growth and survival of tumors with lipogenic phenotypes [38]. Previous studies reported FA can inhibit FASN expression to attenuate lipid accumulation in 3T3-L1 cells [39]. Consistently, this study found that FA decreased lipid content by downregulating FASN protein expression in both vivo and vitro iron overload conditions.

Bile acids, metabolized from cholesterol, exert cytotoxic effects when over-accumulated in liver cells and tissues, contributing to the pathogenesis of fatty liver disease, diabetes, dyslipidemia, and cardiovascular diseases [35]. CYP7A1 is a crucial enzyme that catalyzes the initial and rate-limiting step in the classical bile acid biosynthesis pathway [18]. Notably, previous studies have reported FA could attenuate high-fat diet-induced hypercholesterolemia by activating CYP7A1 to promote bile acid synthesis [20]. However, in the present study, FA inhibited bile acid synthesis through downregulation of CYP7A1. This discrepancy suggests that the regulatory effects of FA on CYP7A1 and bile acid synthesis might be context dependent. Specifically, in our study, we observed that iron overload led to bile acid deposition in the liver to induce hepatotoxicity. In response, FA downregulated CYP7A1, thereby reducing bile acid synthesis. This downregulation can be interpreted as a protective mechanism to mitigate further liver damage by preventing excessive bile acid accumulation. Farnesoid X receptor (FXR) is a bile acid receptor that inhibits CYP7A1 to reduce BA synthesis. In this study, FA upregulated FXR expression in mouse liver but had no significant effect on AML12 cells, suggesting that FA directly regulated CYP7A1 independently of FXR.

The iron-chelating ability of FA plays a crucial role in modulating the expression and activities of FASN and CYP7A1, primarily through its effects on oxidative stress and iron homeostasis. FASN is regulated by oxidative stress levels in the body. ROS can activate FASN and induce fatty acid synthesis [40]. Ferrous iron can promote ROS accumulation via Fenton reactions [41], exacerbating oxidative stress. Ferulic acid, through its iron-chelating ability, can inhibit ROS production and improve antioxidant enzyme activity by activating the Keap1-Nrf2 signaling pathway [42]. By chelating excess iron, FA can reduce the ROS, further inhibiting the expression and activity of FASN. CYP7A1, a member of the cytochrome P450 family, is a heme-containing monooxygenase. The iron ion presented within the heme group plays an essential role in its catalytic activity, participating in the processes of oxygen activation and substrate hydroxylation [43]. We reasonably speculated that the iron-chelating property of FA affects CYP7A1 activity through two mechanisms. Firstly, FA might directly affect the availability of iron needed for heme synthesis, which could subsequently impact the catalytic functionality of CYP7A1. Secondly, the expression and activity of CYP7A1 are regulated by the levels of oxidative stress, with ROS stimulating CYP7A1 and promoting bile acid synthesis [44]. By chelating iron, FA diminishes ROS levels, thereby indirectly inhibiting the expression and activity of CYP7A1.

Collectively, the present study suggests that FA alleviates iron-induced lipid and bile acid metabolism disorders by targeting FASN and CYP7A1.

## 5. Conclusions

In conclusion, using both in vivo and in vitro models, this study demonstrated that iron overload induced disruptions in lipid and bile acid metabolism. We also found FA could alleviate liver injury and reduce iron deposition in mice subjected to iron overload. Furthermore, FA was found to mitigate lipid and bile acid metabolism disorders caused by iron overload by targeting FASN and CYP7A1. Consequently, FA, as a dietary supplement, can potentially prevent and treat chronic liver diseases associated with iron overload by regulating lipid and bile acid metabolism.

## Data Availability

The original contributions presented in the study are included in the article/Supplementary Material, further inquiries can be directed to the corresponding author.

## Supplementary Materials

The following supporting information can be downloaded at: https://www.mdpi.com/article/10.3390/antiox13111277/s1, Supplementary Table S1. Primer sequences and accession numbers of genes used in qRT-PCR. Supplementary Table S2. Details of antibodies. Supplementary Figure S1. Effects of iron overload on oxidative stress, apoptosis, and genomic stability in AML12. Supplementary Figure S2. Effects of iron overload on cell cycle in AML12.

## References

[1] Ganz T. (2013). Systemic iron homeostasis. Physiol. Rev..

[2] Milic S., Mikolasevic I., Orlic L., Devcic E., Starcevic-Cizmarevic N., Stimac D., Kapovic M., Ristic S. (2016). The Role of Iron and Iron Overload in Chronic Liver Disease. Med Sci. Monit..

[3] Britton L., Bridle K., Reiling J., Santrampurwala N., Wockner L., Ching H., Stuart K., Subramaniam V.N., Jeffrey G., St Pierre T. (2018). Hepatic iron concentration correlates with insulin sensitivity in nonalcoholic fatty liver disease. Hepatol. Commun..

[4] Liang Y., Zhang Z., Tu J., Wang Z., Gao X., Deng K., El-Samahy M.A., You P., Fan Y., Wang F. (2021). Gamma-Linolenic Acid Prevents Lipid Metabolism Disorder in Palmitic Acid-Treated Alpha Mouse Liver-12 Cells by Balancing Autophagy and Apoptosis via the LKB1-AMPK-mTOR Pathway. J. Agric. Food Chem..

[5] Frau M., Feo F., Pascale R.M. (2013). Pleiotropic effects of methionine adenosyltransferases deregulation as determinants of liver cancer progression and prognosis. J. Hepatol..

[6] Rahman I., Biswas S.K., Kirkham P.A. (2006). Regulation of inflammation and redox signaling by dietary polyphenols. Biochem. Pharmacol..

[7] Zdunska K., Dana A., Kolodziejczak A., Rotsztejn H. (2018). Antioxidant Properties of Ferulic Acid and Its Possible Application. Ski. Pharmacol. Physiol..

[8] Aalikhani M., Khalili M., Jahanshahi M. (2022). The natural iron chelators’ ferulic acid and caffeic acid rescue mice’s brains from side effects of iron overload. Front. Neurol..

[9] Kose T., Sharp P.A., Latunde-Dada G.O. (2023). Phenolic Acids Rescue Iron-Induced Damage in Murine Pancreatic Cells and Tissues. Molecules.

[10] Sanjeev S., Bidanchi R.M., Murthy M.K., Gurusubramanian G., Roy V.K. (2019). Influence of ferulic acid consumption in ameliorating the cadmium-induced liver and renal oxidative damage in rats. Environ. Sci. Pollut. Res. Int..

[11] Senaphan K., Kukongviriyapan U., Sangartit W., Pakdeechote P., Pannangpetch P., Prachaney P., Greenwald S.E., Kukongviriyapan V. (2015). Ferulic Acid Alleviates Changes in a Rat Model of Metabolic Syndrome Induced by High-Carbohydrate, High-Fat Diet. Nutrients.

[12] Gu Y., Zhang Y., Li M., Huang Z., Jiang J., Chen Y., Chen J., Jia Y., Zhang L., Zhou F. (2021). Ferulic Acid Ameliorates Atherosclerotic Injury by Modulating Gut Microbiota and Lipid Metabolism. Front. Pharmacol..

[13] Wang Z., Yang Y., Zhang J., Hu J., Yan X., Zeng S., Huang X., Lin S. (2021). Ferulic acid ameliorates intrahepatic triglyceride accumulation in vitro but not in high fat diet-fed C57BL/6 mice. Food Chem. Toxicol..

[14] Son M.J., Rico C.W., Nam S.H., Kang M.Y. (2010). Influence of oryzanol and ferulic Acid on the lipid metabolism and antioxidative status in high fat-fed mice. J. Clin. Biochem. Nutr..

[15] Xiong H., Zhang C., Han L., Xu T., Saeed K., Han J., Liu J., Klaassen C.D., Gonzalez F.J., Lu Y. (2022). Suppressed farnesoid X receptor by iron overload in mice and humans potentiates iron-induced hepatotoxicity. Hepatology.

[16] Ocvirk S., O’Keefe S.J.D. (2021). Dietary fat, bile acid metabolism and colorectal cancer. Semin. Cancer Biol..

[17] Lefebvre P., Cariou B., Lien F., Kuipers F., Staels B. (2009). Role of bile acids and bile acid receptors in metabolic regulation. Physiol. Rev..

[18] Fang S., Suh J.M., Reilly S.M., Yu E., Osborn O., Lackey D., Yoshihara E., Perino A., Jacinto S., Lukasheva Y. (2015). Intestinal FXR agonism promotes adipose tissue browning and reduces obesity and insulin resistance. Nat. Med..

[19] Pullinger C.R., Eng C., Salen G., Shefer S., Batta A.K., Erickson S.K., Verhagen A., Rivera C.R., Mulvihill S.J., Malloy M.J. (2002). Human cholesterol 7alpha-hydroxylase (CYP7A1) deficiency has a hypercholesterolemic phenotype. J. Clin. Investig..

[20] Luo Z., Li M., Yang J., Li J., Zhang Y., Liu F., El-Omar E., Han L., Bian J., Gong L. (2022). Ferulic acid attenuates high-fat diet-induced hypercholesterolemia by activating classic bile acid synthesis pathway. Front. Nutr..

[21] Messner D.J., Sivam G., Kowdley K.V. (2009). Curcumin reduces the toxic effects of iron loading in rat liver epithelial cells. Liver Int..

[22] Maslov A.Y., Ganapathi S., Westerhof M., Quispe-Tintaya W., White R.R., Van Houten B., Reiling E., Dolle M.E., van Steeg H., Hasty P. (2013). DNA damage in normally and prematurely aged mice. Aging Cell.

[23] Mah L.J., El-Osta A., Karagiannis T.C. (2010). gammaH2AX: A sensitive molecular marker of DNA damage and repair. Leukemia.

[24] Bai P. (2015). Biology of Poly(ADP-Ribose) Polymerases: The Factotums of Cell Maintenance. Mol. Cell.

[25] Srinivas U.S., Tan B.W.Q., Vellayappan B.A., Jeyasekharan A.D. (2019). ROS and the DNA damage response in cancer. Redox Biol..

[26] Li T., Chiang J.Y. (2014). Bile acid signaling in metabolic disease and drug therapy. Pharmacol. Rev..

[27] Batts K.P. (2007). Iron overload syndromes and the liver. Mod. Pathol..

[28] Wu J.C., Merlino G., Fausto N. (1994). Establishment and characterization of differentiated, nontransformed hepatocyte cell lines derived from mice transgenic for transforming growth factor alpha. Proc. Natl. Acad. Sci. USA.

[29] Hsiao P.J., Chiou H.C., Jiang H.J., Lee M.Y., Hsieh T.J., Kuo K.K. (2017). Pioglitazone Enhances Cytosolic Lipolysis, beta-oxidation and Autophagy to Ameliorate Hepatic Steatosis. Sci. Rep..

[30] Guo L., Zhou S.R., Wei X.B., Liu Y., Chang X.X., Liu Y., Ge X., Dou X., Huang H.Y., Qian S.W. (2016). Acetylation of Mitochondrial Trifunctional Protein alpha-Subunit Enhances Its Stability to Promote Fatty Acid Oxidation and Is Decreased in Nonalcoholic Fatty Liver Disease. Mol. Cell. Biol..

[31] Gao R., Li Y., Xu Z., Zhang F., Xu J., Hu Y., Yin J., Yang K., Sun L., Wang Q. (2023). Mitochondrial pyruvate carrier 1 regulates fatty acid synthase lactylation and mediates treatment of nonalcoholic fatty liver disease. Hepatology.

[32] Choi J.S., Koh I.U., Lee H.J., Kim W.H., Song J. (2013). Effects of excess dietary iron and fat on glucose and lipid metabolism. J. Nutr. Biochem..

[33] Bechmann L.P., Hannivoort R.A., Gerken G., Hotamisligil G.S., Trauner M., Canbay A. (2012). The interaction of hepatic lipid and glucose metabolism in liver diseases. J. Hepatol..

[34] Prasnicka A., Lastuvkova H., Alaei Faradonbeh F., Cermanova J., Hroch M., Mokry J., Dolezelova E., Pavek P., Zizalova K., Vitek L. (2019). Iron overload reduces synthesis and elimination of bile acids in rat liver. Sci. Rep..

[35] Chiang J.Y. (2013). Bile acid metabolism and signaling. Compr. Physiol..

[36] Cheng Q., Li Y.W., Yang C.F., Zhong Y.J., He H., Zhu F.C., Li L. (2018). Methyl ferulic acid attenuates ethanol-induced hepatic steatosis by regulating AMPK and FoxO1 Pathways in Rats and L-02 cells. Chem. Biol. Interact..

[37] Lambruschini C., Demori I., El Rashed Z., Rovegno L., Canessa E., Cortese K., Grasselli E., Moni L. (2020). Synthesis, Photoisomerization, Antioxidant Activity, and Lipid-Lowering Effect of Ferulic Acid and Feruloyl Amides. Molecules.

[38] Fhu C.W., Ali A. (2020). Fatty Acid Synthase: An Emerging Target in Cancer. Molecules.

[39] Koh E.J., Kim K.J., Seo Y.J., Choi J., Lee B.Y. (2017). Modulation of HO-1 by Ferulic Acid Attenuates Adipocyte Differentiation in 3T3-L1 Cells. Molecules.

[40] He M., Li X., Yu L., Deng S., Gu N., Li L., Jia J., Li B. (2022). Double-Sided Nano-ZnO: Superior Antibacterial Properties and Induced Hepatotoxicity in Zebrafish Embryos. Toxics.

[41] Xing G., Meng L., Cao S., Liu S., Wu J., Li Q., Huang W., Zhang L. (2022). PPARalpha alleviates iron overload-induced ferroptosis in mouse liver. EMBO Rep..

[42] Zhang Z., Yang P., Zhao J. (2022). Ferulic acid mediates prebiotic responses of cereal-derived arabinoxylans on host health. Anim. Nutr..

[43] Yu Cai Lim M., Kiat Ho H. (2024). Pharmacological modulation of cholesterol 7alpha-hydroxylase (CYP7A1) as a therapeutic strategy for hypercholesterolemia. Biochem. Pharmacol..

[44] Gonzalez R., Cruz A., Ferrin G., Lopez-Cillero P., Fernandez-Rodriguez R., Briceno J., Gomez M.A., Rufian S., Mata Mde L., Martinez-Ruiz A. (2011). Nitric oxide mimics transcriptional and post-translational regulation during alpha-tocopherol cytoprotection against glycochenodeoxycholate-induced cell death in hepatocytes. J. Hepatol..

