# Supplementary material for: Ferulic Acid Alleviates Lipid and Bile Acid Metabolism Disorders by Targeting FASN and CYP7A1 in Iron Overload-Treated Mice
Source: Antioxidants (Basel). 2024 Oct 23;13(11):1277. doi: 10.3390/antiox13111277 (PMC11591460; doi:10.3390/antiox13111277)
Supplement: Supplementary file 1 [file antioxidants-13-01277-s001.zip › antioxidants-3194847-supplementary.pdf]

Supplementary Table S1. Primer sequences and accession numbers of genes used in qRT-PCR.

Gene	Primer sequences (5′-3′)	Accession numbes
Gapdh	F: GGCACAGTCAAGGCTGAGAATG<br>R: ATGGTGGTGAAGACGCCAGTA	NM 001289726.1
Bax	F: CCAGGGTGGCTGGGAAG<br>R: CTTCCAGATGGTGAGCGAGG	NM_007527.3
Bcl2	F: CGTCGTGACTTCGCAGAGAT<br>R: TAGTTCCACAAAGGCATCCCAG	NM_009741.5
Caspase3	F: GAGCTTGGAACGGTACGCTA<br>R: CCGTACCAGAGCGAGATGAC	NM_001284409.1
Fasn	F: ACACATCCTAGGCATCCGAG<br>R: TTTCCTGAAGTTTCCGCAGC	NM_007988.3
Acc	F: CCGTTGGCCAAAACTCTGGAGCTAA<br>R: GAGCTGACGGAGGCTGGTGACA	XM_030245463.1
Srebf	F: GGAACTTTTCCTTAACGTGGGC<br>R: AGCTGGAGCATGTCTTCGAT	NM_001313979.1
Pparα	F: GTGCAGCCTCAGCCAAGTT<br>R: TGGGGAGAGAGGACAGATGG	NM_001113418.1
Acox1	F: GATAAACTCCCCAAGATTCAA<br>R: AAGTCAAAGGCATCCACCAAA	NM015729.2
Adipoq	F: GCTCCTGCTTTGGTCCCTCCAC<br>R: GCCCTTCAGCTCCTGTCATTCC	NM_009605.4
Cpt1α	F: GACTCCGCTCGCTCATTCC<br>R: GGCAGATCTGTTTGAGGGCT	NM_013495.2
Cyp7a1	F: CAACGGGTTGATTCCATACC<br>R: ATTTCCCCATCAGTTTGCAG	NM_007824.3
Bsep	F: GGACAATGATGTGCTTGTGG<br>R: CACACAAAGCCCCTACCAGT	NM_001363492.1
Fxr	F: CAGAAATGGCAACCAGTCATGTA<br>R: AAATCTCCGCCGAACGAA	XM_030244963.2
Shp	F: CTCATGGCCTCTACCCTCAA<br>R: GGTCACCTCAGCAAAAGCAT	NM_011850.3
Hamp	F: AGGGCAGACATTGCGATACC<br>R: GCAACAGATACCACACTGGGA	NM_032541.2
Dmt1	F: TGGCTCCTGGGATATGGAGT<br>R: TGCTGTAGGCAGGGTTGATG	NM_001356952.1
Tfrc	F: TCATGAGGGAAATCAATGATC<br>R: GCCCCAGAAGATATGTCGGAA	XM_036159875.1
Slc39a14	F: TTTCCCAGCCCAAGGAAG<br>R: CAAAGAGGTCTCCAGAGCTAAA	XM_030247713.2

Fpn	F: ATGGGAACTGTGGCCTTCAC<br>R: TCCAGGCATGAATACGGAGA	XM_006496137.4

Supplementary Table S2. Details of antibodies.

Antibodies	Cat No.	Source	Dilution
GAPDH	60004-1	Proteintech, Chicago, USA	1:50000 (WB)
FTH	Bs-8679R	Bioss, Beijing, China	1:1000 (WB)
KEAP1	10503-2-AP	Proteintech, Chicago, USA	1:5000 (WB)
HO-1	10701-1-AP	Proteintech, Chicago, USA	1:3000 (WB)
BAX	50599-2-lg	Proteintech, Chicago, USA	1:5000 (WB)
BCL2	26593-1-AP	Proteintech, Chicago, USA	1:2000 (WB)
PARP1	13371-1-AP	Proteintech, Chicago, USA	1:1000 (WB)
PRDX2	10545-2-AP	Proteintech, Chicago, USA	1:5000 (WB)
γH2A.X	2577	CST, Beverly, MA, USA	1:400 (IF)
CPT1A	15184-1--AP	Proteintech, Chicago, USA	1:5000 (WB)
PPARA	15540-1-AP	Proteintech, Chicago, USA	1:1000 (WB)
FASN	10624-2-AP	Proteintech, Chicago, USA	1:5000 (WB)
CYP7A1	DF2612	Affinity, Cincinnati, OH, USA	1:1000 (WB)
FXR	A12788	Abclonal	1:1000 (WB)
BSEP	DF9278	Affinity, Cincinnati, OH, USA	1:1000 (WB)
Anti-rabbit IgG(H+L)	SA00001-2	Proteintech, Chicago, USA	1:4000 (WB)
HRP-labeled Goat Anti-Mouse IgG(H+L)	SA00001-1	Proteintech, Chicago, USA	1:4000 (WB)

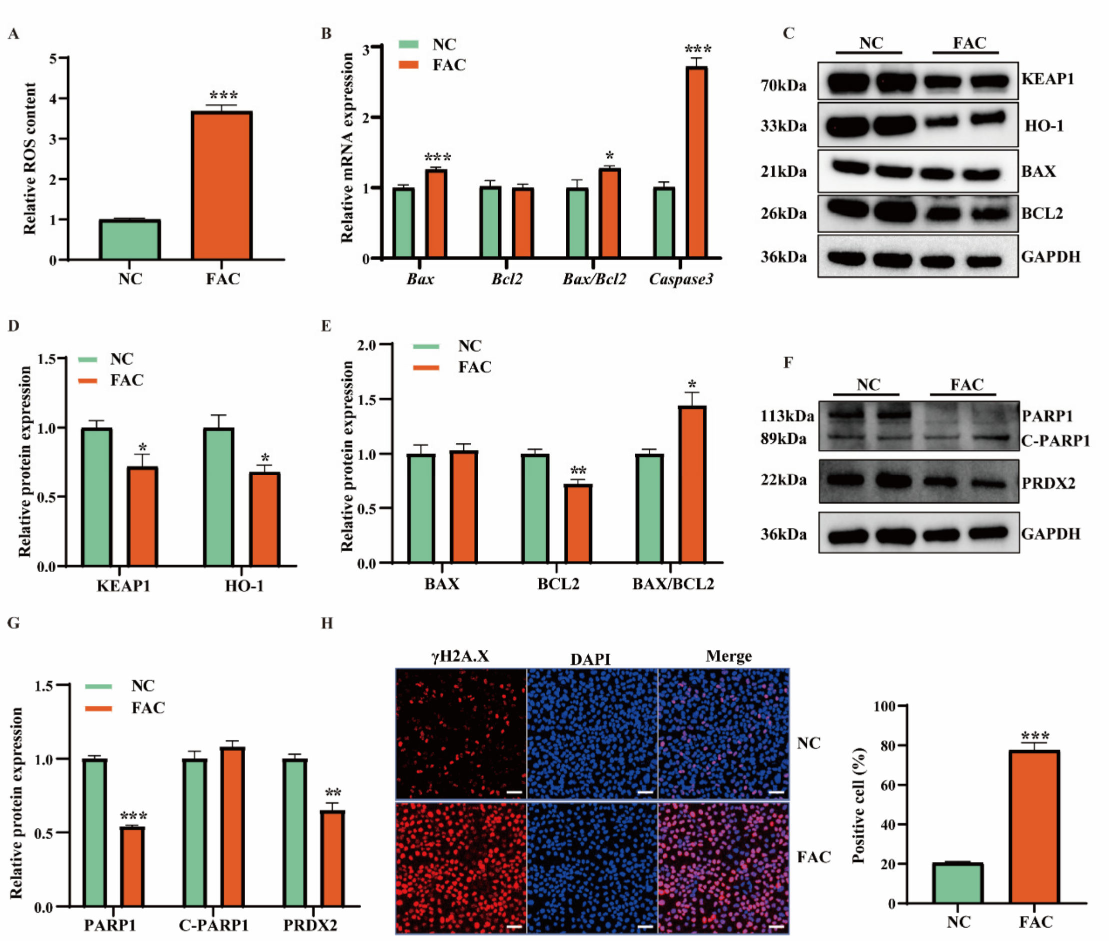

Figure S1. Effects of iron overload on oxidative stress, apoptosis, and genomic stability in AML12.

A: Relative ROS content in AML12 cells treated with or without FAC.

B: qRT-PCR analysis of apoptosis-related genes.

C-E: Western blot analysis of antioxidant-related proteins and apoptosis-related proteins.

F-G: Western blot analysis of genomic stability-related proteins.

H: Immunofluorescence analysis of DNA damage marker γH2A.X. Scale bar, 50 μm.

Statistical significance was indicated as $*P < 0.05$, $**P < 0.01$, $***P < 0.001$, determined using a two-tailed t-test.

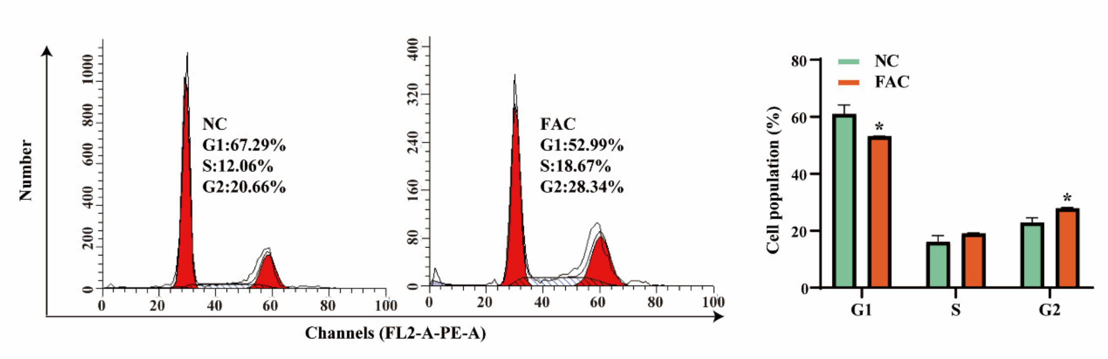

Figure S2. Effects of iron overload on cell cycle in AML12.

The cell cycle distribution in AML12 cells treated with or without FAC was detected by flow cytometry.

Statistical significance was indicated as *$P < 0.05$, determined using a two-tailed t-test.
